# Salivary pH, but not conductivity, is an indicator of diarrhea in neonatal calves

**DOI:** 10.3389/fvets.2024.1483890

**Published:** 2024-12-18

**Authors:** Beth B. Riley, Alexander Corbishley, Marie J. Haskell, Carol-Anne Duthie, Alastair I. Macrae, Elizabeth Burrough, Colin Mason

**Affiliations:** ^1^Scotland’s Rural College (SRUC), Edinburgh, United Kingdom; ^2^Clinical Sciences, The Royal (Dick) School of Veterinary Studies, The University of Edinburgh, Edinburgh, United Kingdom; ^3^Dairy Herd Health and Productivity Service, The Royal (Dick) School of Veterinary Studies, The University of Edinburgh, Edinburgh, United Kingdom; ^4^The Roslin Institute, The University of Edinburgh, Edinburgh, United Kingdom

**Keywords:** biomarker, conductivity, dehydration, hematocrit, neonatal calf diarrhea, pH, saliva, total protein

## Abstract

Neonatal calf diarrhea is a frequent disease of calves and may result in dehydration and metabolic acidosis. The disease causes mortality and reduces growth and future productivity. Early identification of disease improves calf outcomes and thus there is increasing interest in technological methods for detecting disease. Dehydration leads to the blood becoming more concentrated and this can be measured using serum osmolality. Research in humans has shown that saliva conductivity is correlated with serum osmolality. Saliva conductivity may therefore offer a non-invasive opportunity to assess hydration status in calves. Furthermore, as blood pH is a prognostic indicator and there is ion exchange in the salivary ducts, saliva pH may act as an indicator of metabolic acidosis. This observational study aimed to assess the relationship of saliva conductivity and pH with the clinical and biochemical parameters of calves suffering from neonatal calf diarrhea. One hundred and forty-one dairy-bred calves were recruited onto the study at approximately 1 week of age. The health of the calves was assessed daily. Calves had blood and saliva samples taken weekly until 25 days of age or the development of neonatal calf diarrhea. When calves developed diarrhea, they were sampled for three consecutive days. Hematocrit, plasma total protein, saliva pH and saliva conductivity were measured at each sampling. Saliva pH and saliva conductivity were measured using portable meters (*LAQUAtwin-pH-33* and *LAQUAtwin-EC22*). In a subset of 30 matched samples, serum proteins and electrolytes were also measured. Saliva conductivity was not associated with diarrhea or dehydration. Saliva pH was lower in calves with diarrhea, regardless of hydration status. The Lin’s concordance correlation coefficients between saliva variables and hematocrit and strong ion difference were negligible. Dehydrated calves with diarrhea had a higher hematocrit and albumin and the lowest sodium and SID. Calves with diarrhea and no dehydration had a lower plasma total protein. While saliva conductivity has been associated with measures of dehydration in humans, this does not appear to be the case in calves. Saliva pH has not previously been considered for disease detection; however as it is associated with diarrhea, further research is warranted.

## 1 Introduction

Diarrhea is considered by both veterinarians and farmers to be one of the main threats to calf health (1). The prevalence and mortality rates for neonatal calf diarrhea (NCD) are variable in the literature. A UK study of 11 farms found that NCD affects an average of 48.2 per 100 calves in the first 10 weeks of life (2). However, a study of 10 dairy farms in the US found a incidence of 77 per 100 calves in the first 30 days of life, with 1.4% of diarrhea cases dying (3). A larger study in the US found that 17.2% of calves developed NCD in the pre-weaning period, with 8.5% of cases experiencing mortality (4). However, where NCD is recorded by researchers and not farm staff, the disease rates recorded are far higher. A study in the UK recorded an incidence of 64.5 per 100 calves in the first 8 weeks of life (5), a US study recorded a incidence of 85 per 100 calves under 28 days of age across four farms (6) and a Canadian study found an incidence of 97 per 100 calves under 49 days of age on a single farm (7). Together these data suggests that NCD is a widespread problem.

The NCD can cause impaired welfare (8), and has far reaching consequences in calves that have been affected. The number of weeks a dairy calf has diarrhea prior to weaning has been found to be negatively associated with average daily liveweight gain between 1 and 63 days of age (9). Holstein heifer calves which suffered from diarrhea pre-weaning had a lower average daily liveweight gain at weaning, were older at first calving and had a lower 305 day mature equivalent milk yield (10). These calves are also more likely to be removed from the herd in their first 300 days in milk in one study (11) but not in another (12). Beef calves suffering from severe diarrhea in the first 16 days of life were found to gain 34 kg less in the first 6 months of life (13). Furthermore, diarrhea on arrival to a veal unit has been associated with increased risk of disease and mortality during their time at the unit (14) but not with weight gain while at the unit (15). The proportion of days with diarrhea at a veal unit has been associated with a reduction in weight gain (16). A consequence of NCD can be dehydration, which in its severe form increases the risk of mortality (17).

Early detection of NCD and dehydration can lead to improved calf outcomes and reduce the costs associated with treatment, mortality, and reduced growth rates (18). This had led to an interest in technological solutions for disease detection. Existing research includes investigation of ear mounted accelerometers (18, 19), leg mounted accelerometers (20), automatic milk feeders (19, 21, 22) and infrared thermography (23). However, as yet, there is no research on the use of technologies to detect dehydration. Traditionally, dehydration is evaluated by using blood samples to measure the hematocrit (the percentage by volume of red blood cells in blood) and plasma total protein (PTP) in the laboratory. This is both invasive for the calf and means that there is a time lag before results are available to the farmer or veterinary surgeon. There is therefore an opportunity to explore other parameters that can be measured in a non-invasive manner. This study aimed to look at measurements from saliva that could be measured using a portable meter.

Saliva osmolality has been shown to increase with dehydration in humans (24). One study has demonstrated that saliva osmolality increases linearly with body mass loss (a measure of dehydration) in dehydrated humans (25), although another study suggested that this relationship was non-linear (26). The diagnostic accuracy (receiver-operating characteristic-area under the curve) of saliva osmolality for mild intracellular dehydration in humans was 0.70 (confidence interval 0.51–0.85) (27). Saliva conductivity is correlated with serum osmolality in humans (28) and thus has potential to be used to monitor dehydration, especially as conductivity can be measured with a portable meter. Previous research into calf saliva has included measuring saliva cortisol for stress (29), and saliva immunoglobulins to monitor for failure of passive transfer (30). However, spectrophotometric methods such as enzyme-linked immunosorbent assays are required to measure all these analytes, and this requires a diagnostic laboratory.

The NCD also frequently results in metabolic acidosis (31). It is thought that this is due to the production of D-lactate in the colon (31). and sodium loss through the gastrointestinal tract (32). In young calves, metabolic acidosis is associated with an increased risk of mortality (33). Blood gas analysis has been considered beneficial for assessment of diarrhea in calves (32) as it gives a more detailed assessment of the nature of the electrolyte imbalances present (34). However, the equipment required is costly (34). It has been previously suggested that the pH of whole blood could be an alternative to blood gas analysis, due to the high correlation between blood pH and a clinical assessment scoring system (34). Blood pH has also been shown to be a predictor for mortality (17). However, a test that can be done pen-side would allow more immediate treatment, while minimizing invasive procedures on sick calves will reduce stress. Furthermore, there is potential for the development of systems to collect calf saliva to examine the health status of a group as has previously been suggested in pigs (35) or possibly an individual if measurement at the teat is possible. If individual identification of dehydrated calves is possible at an early stage this would allow earlier intervention and thus improve welfare. Saliva pH has previously been measured in calves when examining the development of the rumen (36). Thus, saliva pH could potentially be a tool for the detection of metabolic acidosis in calves.

This observational study aimed to assess the relationship of saliva conductivity and pH with the clinical and biochemical parameters of calves suffering from NCD.

## 2 Materials and methods

### 2.1 Animal management

The animal work for this study was conducted at the SRUC Dairy Research and Innovation Centre, Crichton Royal Farm, Dumfries, UK under the approval of the Animal Experiment Committee of SRUC (DAI AE 06–2022). One hundred and forty-one dairy-bred calves were recruited from the all year round calving Holstein herd, and managed under the normal rearing conditions of the farm. This sample size was based on the previous understanding of NCD prevalence on the farm. Each calf received 4 L of defrosted cow’s colostrum by stomach tube within 8 h of birth. Calves were housed in straw or woodchip bedded individual hutches from birth to approximately 7 days of age, where they were fed six liters of milk replacer split between two meals daily (*Maximum +, Carrs Billington Agriculture (Sales) Ltd., Carlisle*, crude protein 24%, crude oils and fats 20%, crude ash 7.5%, calcium 0.9%, sodium 0.5%, phosphorus 0.7%, 176 g/L). Calves had *ad-libitum* access to concentrate pellets (*Ambition calf and Omnigen nuts*, *Mole Valley Feed Solutions, South Molton*, dry matter 86.2%, crude protein 18%, crude oils and fats 4.6%, crude fiber 9.2%, crude ash 9.2%, Sodium 0.5%. Selenium 0.4 mg/kg, copper 19 mg/kg, Vitamin E 60 mg/kg) and water. Due to a history of severe acute cryptosporidiosis in calves less than 7 days of age, calves received paromomycin sulfate in milk for their time in the individual hutches (*Parofor, Huvepharma NV, Antwerp, Belgium*, 10 g/calf/day) as advised by the farm’s independent veterinary surgeons.

Calves were transferred to group pens containing 10–12 animals at approximately 7 days of age. Group pens were straw-bedded and consisted of an igloo (3.9 m × 4.4 m, 2.2 m high) and an adjacent covered pen (5.1 m × 5.1 m) (stocking density 3.6–4.3 m^2^/calf). Each calf had access to seven liters of milk replacer daily (as above) using an automatic milk feeder (*custom built for this calf unit, BioControl Norway As, Grimstad Gård, Norway*). Calves had *ad libitum* access to water, concentrate (as above) and straw.

### 2.2 Health assessment

Calves were recruited to the study 24 h after entry into the group pen. Health scoring was carried out daily, including the Wisconsin calf health score (HEALTH) (37), a long-established tool that includes temperature, ocular and nasal discharge, ear posture, and cough. In addition, tail, perineum and hind leg cleanliness (CLEAN) and feces were scored on a scale of 0–3. The scoring system for both CLEAN and the feces score are shown in Table 1. Feces was scored whenever defecation was observed while in the pen for scoring. Skin tent elasticity and capillary refill time were recorded to determine whether the calf was suffering from dehydration. Skin tent elasticity was measured behind the shoulder. A calf was designated as being dehydrated when the rebound of the skin tent took more than 3 s. Capillary refill time was measured using the oral mucosa. These measures were chosen as they were easy to train and were both associated with a >4% reduction in hydration in young calves when over 3 s (38). Additional criteria were needed as HEALTH does not consider dehydration and in group housed calves feces can only be linked to an individual if defecation is observed. NCD was classified as a feces score of ≥2 or a CLEAN score of ≥2. Scoring was predominantly carried out by one person (BR, 85%) with the remainder being carried out by three research technicians. All four scorers trained together and compared until consistent.

**Table 1:** The criteria for the tail, perineum and hind leg cleanliness (CLEAN) and feces scores.

	Scoring criteria	
Score	CLEAN	Feces (68)
0	Clean calf or with a small amount of dried feces on tail/perineum/hind legs	Formed feces
1	A large amount of dried feces or some pasty feces on tail/perineum/hind legs	Pasty feces
2	Wet feces on tail/perineum/hind legs	Loose feces that did not sift through bedding
3	A very wet tail/perineum or a large amount of feces on tail/perineum/hind legs	Liquid feces that sifted through the bedding

Disease was not induced in this study. Where the research team detected spontaneously occurring disease, it was reported to the farm manager based on the following pre-agreed severity criteria: low level of milk consumed in a calf with NCD (<2 L by 10:30 am), depressed calf demeanor, fever, Wisconsin score >2 or dehydration. Calves were treated according to protocols agreed with the veterinary surgeon responsible for the farm. This was a non-steroidal anti-inflammatory for all calves (meloxicam, *Meloxidyl 20 mg/mL solution for injection for cattle, pigs and horses, CEVA Animal Health Ltd., Woodburn Green, Buckinghamshire*, 0.5 mg/kg subcutaneously), a 3 day course of antibiotic for those with a fever or blood in the feces (trimethoprim and sulfadiazine, *Norodine 24 solution for injection, Norbrook, Newry, County Down*, 15 mg/kg intramuscularly) and oral rehydration for dehydrated/dull calves (*Life-Aid Xtra, Norodine 24 solution for injection, Norbrook, Newry, County Down*, mixed with 2 L water and administered by stomach tube).

### 2.3 Samples

Saliva and blood samples were taken from healthy calves on the day of recruitment and weekly thereafter (“non-diseased” samples). When NCD was detected from feces or CLEAN score, saliva and blood samples were taken on the day of detection and for two subsequent days (“diseased” samples). Fecal samples were also taken on the first day that NCD was detected. Calves finished the trial at 26 days of age or after one episode of NCD.

#### 2.3.1 Feces

Each feces sample was tested using an immunochromatography tests for rotavirus, cryptosporidium, *E. coli* K99 and bovine coronavirus (*Expertis scour check test*, *MSD Animal Health, Walton, Milton Keynes* or *Surecheck 4, Nimrod Veterinary Products Ltd., Moreton-in-Marsh Gloucestershire*) according to the provided instructions.

#### 2.3.2 Saliva

Saliva was sampled using a Salivette*^®^* sponge (*Salivette^®^, SARSTEDT AG & Co. KG, Sarstedtstraße, Nümbrecht, Germany*) held in a pair of forceps, which was placed in the calf’s mouth for 1 min. This was based on experience during a pilot trial to allow sufficient volume to be collected. The sponge was then placed in the top portion of the Salivette tube. The forceps were disinfected between calves.

The Salivette tubes were centrifuged at 981 *g* for 15 min and the sponge and top portion of the tube removed. The saliva conductivity was measured by placing approximately one-sixth of the sample on the sensor of the conductivity meter (*LAQUAtwin-EC-22, HORIBA Advanced Techno Co. Ltd., Kisshoin Minami-ku, Kyoto, Japan*). Each sample was measured three times and the mean calculated. The saliva pH was measured in the same way using a pH meter (*LAQUAtwin-pH-33, HORIBA Advanced Techno Co. Ltd., Kisshoin Minami-ku, Kyoto, Japan*). Saliva was predominately analyzed within 8 h, if analysis within 30 h was not possible then saliva was frozen in aliquots at −20°C. There was no difference between the pH of fresh and defrosted saliva (results not shown).

#### 2.3.3 Blood

Blood samples were taken from the jugular vein. The hair covering the jugular groove was clipped on entry to the study. Venipuncture was performed and 6 mL of blood was taken into a clot activator tube (*VACUETTE^®^ 6 mL CAT Serum, Greiner Bio-One Ltd., Stonehouse, Gloucestershire*) and a further 4 mL into a potassium Ethylenediaminetetraacetic (EDTA) tube (*VACUETTE^®^ 4 mL K2EDTA, Greiner Bio-One Ltd., Stonehouse, Gloucestershire*). Serum was collected by centrifuging the clot activator tube at 981 *g* for 10 min and removing the serum using a Pasteur pipette. This was then frozen at −20°C for subsequent analysis.

Hematocrit was measured by centrifuging (3 min at 13,000 *g*) two capillary tubes of whole EDTA blood per sample. These were then measured using a hematocrit reader (39) and the mean calculated. PTP was measured by centrifuging whole EDTA blood (10 min at 981 *g*) and then pipetting the plasma on to a refractometer (*RHC-200/ATC, Mag-Tek Dual Scale Refractometer, Gain Express Holdings Ltd., To Kwa Wan, Kowloon, Hong Kong*). Each sample was measured twice and the mean calculated (39).

### 2.4 Data processing and analysis

All data were recorded in Microsoft Excel and data processing was carried out using the tidyverse package (40) in R (41) using the R studio graphical interface (*R studio, Boston, Massachusetts*). Any non-diseased samples taken within 2 days preceding the development of NCD or on a day where the HEALTH score was >4 (classed as intermediate or diseased) were excluded from all further analysis. The remaining non-diseased samples were classified as “healthy.” The diseased samples were sub-classified according to the hydration status of the calf on the day of sampling. Specifically, samples taken on a day where the skin tent elasticity was normal were classified as Neonatal Calf Diarrhea Hydrated (NCD-H) and samples taken on a day where the skin tent return was delayed were classified as Neonatal Calf Diarrhea Dehydrated (NCD-D).

To test the hypotheses that saliva conductivity, saliva pH, PTP and hematocrit were associated with NCD and dehydration, general linear mixed models were constructed for each of these parameters in turn. The calf identity nested within group was used as a random effect in all models. Disease status (Healthy, NCD-H or NCD-D), sex, sire breed-type, the interaction between sex and sire breed-type, age, the interaction between disease and age, the interaction between age and sex, age at inclusion into the group pen, and date were all included in the maximal model. The step() function [stats package, (41)] was used to carry out backwards model selection using the Akaike information criterion (AIC). The final model was checked using the simulate residual() and plotQQunif() functions in the DHARMa package (42). Where appropriate, the response variable was transformed. A natural log transformation was used on the saliva conductivity and a square transformation was used on saliva pH. The model output was calculated using the summary () (43) and confint() functions (41) with the anova() function (41) used to calculate the numerator degrees of freedom (NDF) and denominator degrees of freedom (DDF). The estimated marginal means and pairwise comparisons were calculated from the model using the emmeans() function in the emmeans package (44), and plotted using ggplot2 (45).

In order to further explore the potential of saliva parameters to detect dehydration, correlations between hematocrit (a hematological proxy for dehydration) and saliva pH and conductivity were tested. Scatter diagrams were plotted using ggplot2 (45). Lin’s correlation concordance coefficient (pC) were tested using the CCC() function in the DescTools package (46).

#### 2.4.1 Serum biochemistry

To examine the relationship between saliva parameters, metabolic acidosis and serum proteins in calves with diarrhea, a subset of samples were selected. A balanced dataset was created by matching each calf with NCD-D with a healthy and NCD-H calf on the following basis: date of sampling (within 14 days), age (within 7 days), sire breed-type, sex, and disease stage. Each calf was used only once. The matching criteria were chosen based on the results of the healthy calf analysis (Supplementary material) and diseased models.

Sodium, potassium, chloride, serum total protein (STP) and albumin were measured in defrosted serum samples using an AU80 Chemistry Analyzer with ISE unit (*Beckman Coulter Ireland Inc., Lismeehan, O’Callaghans Mills, Co. Clare, Ireland*). Globulin was calculated by the analyzer from the albumin and STP. The strong ion difference (SID, a proxy for metabolic acidosis) was calculated as below (47).


$$SID=\left(\text{Sodium}+\text{Potassium}\right)-\text{Chloride}$$

IgG was measured using radial-immunodiffusion, following the manufacturer’s instruction using kits provided by SCCL (*SCCL, 30 Molaro Place, Saskatoon, SK, Canada, S7K 6A2*).

Linear models were built for each of hematocrit, saliva conductivity, saliva pH, STP, albumin, globulin, potassium, sodium, chloride and SIG using the lm() function in the stats package (41). Disease status (Healthy, NCD-H or NCD-D), age, sex, sire breed-type, season, age at inclusion and the interaction between age and disease status were tested as fixed effects. The step procedure in the stats package (41) was used to perform backwards model selection. The selected model was then checked using the simulateresiduals() and plotqqunif() functions in the DHARMa package (42). The outputs of the models were calculated using the summary(), anova() and confint() functions (41). The estimated marginal means were calculated using the emmeans() function in the emmeans package (44) and plotted using ggplot2 (45).

In order to further explore the potential of saliva parameters to detect metabolic acidosis in calves, correlations between SID (a proxy for acidosis) and saliva pH and conductivity were explored. Scatter diagrams were plotted using ggplot2 (45). pC was tested using the CCC() function in the DescTools package (46).

## 3 Results

Of the 141 calves recruited onto the study, 108 developed NCD. Ninety-eight of these did not develop dehydration and 10 of these cases developed dehydration. Each dehydrated calf was only dehydrated for 1 day.

### 3.1 Pathogens

All 108 calves that developed NCD had a feces sample tested. Of these 49 were positive for Cryptosporidium, 27 were negative for all four pathogens tested, 14 were positive for Bovine Rotavirus, five were positive for Cryptosporidium, Bovine Rotavirus and Bovine Coronavirus, four were positive for Cryptosporidium and Bovine Rotavirus, three were positive for Cryptosporidium and Bovine Coronavirus, two were positive for Bovine Coronavirus, one was positive for Bovine Rotavirus and Bovine Coronavirus and one was positive for Cryptosporidium, Bovine Rotavirus and *E. coli* K99.

### 3.2 The association of neonatal calf diarrhea and hydration status with blood and saliva variables

There were 488 sampling events from 139 calves available for analysis. One calf was excluded due to developing clostridial abomasitis the day after sampling and another had a HEALTH score >4 at each sampling event so was excluded from analysis. The number of samples in each disease category (Healthy, NCD-H, NCD-D) as well as the sex and sire breed-type of the corresponding calves are shown in Supplementary Table S1. The descriptive statistics of the continuous calf variables, and the saliva and blood parameters from this data set are shown in Supplementary Table S2.

#### 3.2.1 Calves with diarrhea had a lower saliva pH

**Figure 1:**
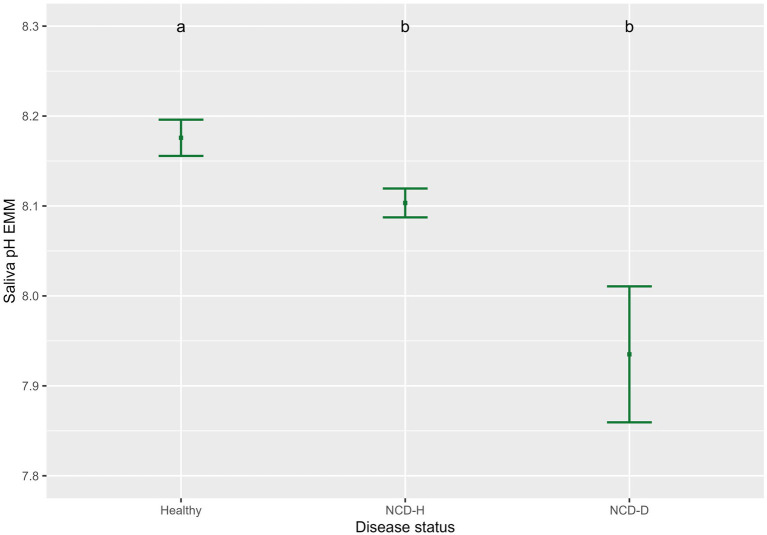
Association of neonatal calf diarrhea and hydration status with saliva pH. Plots and error bars denote the estimated marginal means (EMM) and corresponding standard errors calculated from the final model. Differing letters indicate statistically significant differences. Healthy: 164 calf days (105 calves), NCD-H: neonatal calf diarrhea-hydration status normal: 314 calf days (109 calves); NCD-D: neonatal calf diarrhea-dehydrated: 10 calf days (10 calves).

Saliva conductivity was not associated with disease status (when compared to healthy calves; NCD-H: *p* = 0.12; NCD-D: *p* = 0.692, Table 2). Calves with diarrhea had a lower saliva pH than their healthy counterparts (NCD-H: *p* < 0.01, and NCD-D: *p* < 0.01, Figure 1). Dehydrated calves were not different from NCD-H calves (*p* = 0.07, Figure 1). Calves that were introduced into the group pen at an older age had a higher saliva conductivity (*p* = 0.02, Table 2).

**Table 2:** The association of neonatal calf diarrhea and hydration status with changes in saliva and blood parameters in artificially reared calves.

Variable	Factor	Level	Number of calf days	Estimate	Confidence interval	Numerator degrees of freedom	Denominator degrees of freedom	*t-*value	*p*-value
Saliva conductivity (mS/cm)^a^	Disease	Healthy	164^c^	Reference	Reference	2	453.62	Reference	Reference
		NCD-H^b^	314^d^	1.03	0.99 – 1.08			1.543	0.123
		NCD-D^b^	10^e^	1.03	0.85 – 1.11			−0.397	0.692
	**Age at inclusion into the group pen**		**488** ^**f**^	**1.06**	**1.01–1.10**	**1**	**138.15**	**2.384**	**0.019**
Saliva pH^a^	Disease	Healthy	164^c^	Reference	Reference	2	469.27	Reference	Reference
		**NCD-H**	**314** ^**d**^	**−1.37**	**−1.38 – −0.68**			**−3.196**	**0.002**
		**NCD-D**	**10** ^**e**^	**−1.97**	**−2.51 – −1.20**			**−3.160**	**0.002**
Hematocrit (%)	Disease	Healthy	164^c^	Reference	Reference	2	399.10	Reference	Reference
		NCD-H	314^d^	−0.13	−0.96 – 0.70			−0.307	0.759
		**NCD-D**	**10** ^**e**^	**4.02**	**1.44–6.61**			**3.059**	**0.002**
	Sex	Female	228^g^	Reference	Reference	1	128.51	Reference	Reference
		**Male**	**260** ^**h**^	**−1.84**	**−3.52 – −0.16**			**−2.168**	**0.032**
	Sire breed-type	Beef	393^i^	Reference	Reference	1	133.06	Reference	Reference
		**Dairy**	**95** ^**j**^	**−3.15**	**−5.31 – −0.98**			**−2.893**	**0.005**
Plasma total protein (g/dL)	Disease	Healthy	164^c^	Reference	Reference	2	432.25	Reference	Reference
		**NCD-H**	**314** ^**d**^	**−0.18**	**−0.28 – −0.08**			**−3.590**	**<0.001**
		NCD-D	10^e^	0.08	−0.16 – 0.32			0.620	0.536
	Sire breed-type	Beef	393^i^	Reference	Reference	1	136.05	Reference	Reference
		**Dairy**	**95** ^**j**^	**−0.33**	**−0.55 – −0.11**			**−3.011**	**0.003**
	**Age**		**488** ^**f**^	**−0.03**	**−0.04 – −0.02**	**1**	**472.28**	**−5.697**	**<0.001**

The results shown are from the final linear mixed models. Variables shown in bold have *p* < 0.05.

a Values have been back transformed.

b NCD-H: neonatal calf diarrhea-hydration status normal; NCD-D: neonatal calf diarrhea-dehydrated.

c 105 calves, ^d^109 calves, ^e^10 calves, ^f^139 calves, ^g^75 calves, ^h^64 calves, ^i^112 calves, ^j^27 calves.

#### 3.2.2 Hematocrit was highest in dehydrated calves and protein lowest in NCD-H calves

**Figure 2:**
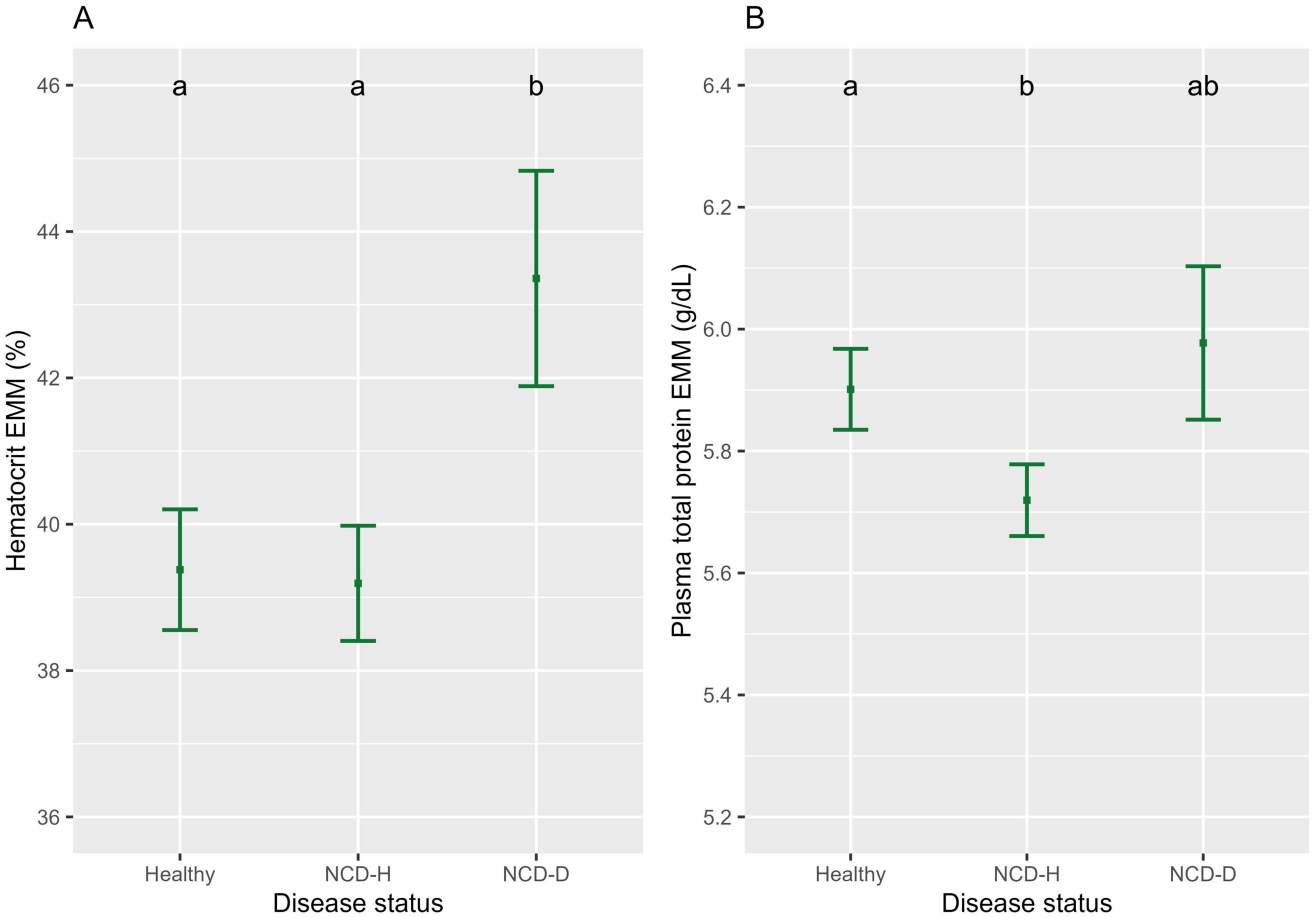
The association of neonatal calf diarrhea and hydration status with **(A)** hematocrit and **(B)** plasma total protein. Plots and error bars denote the estimated marginal means (EMM) and corresponding standard errors calculated from the final models. Differing letters indicate statistically significant differences. Healthy: 164 calf days (105 calves), NCD-H: neonatal calf diarrhea-hydration status normal: 314 calf days (109 calves); NCD-D: neonatal calf diarrhea-dehydrated: 10 calf days (10 calves).

Hematocrit was highest in dehydrated calves (relative to healthy calves, *p* < 0.01, relative to NCD-H calves, *p* < 0.01, Figure 2A). There was no difference between healthy and NCD-H calves (*p* = 0.90, Figure 2A).

NCD-H calves had the lowest PTP when compared to healthy calves (*p* < 0.01, Figure 2B). NCD-D calves were not different from either healthy or NCD-H calves (*p* = 0.81, and *p* = 0.07 respectively, Figure 2B).

Male calves had higher hematocrit than female calves (*p* = 0.03, Table 2). Both hematocrit and PTP were lower in dairy-sired calves: than in their beef-sired counterparts (*p* < 0.01, and *p* < 0.01, respectively, Table 2). PTP was highest in younger calves and declined as calves got older (*p* < 0.001, Table 2).

#### 3.2.3 Saliva parameters were not correlated with hematocrit

Saliva parameters were explored for the Lin’s correlation concordance with hematocrit, which is a proxy for dehydration. The correlation between both saliva pH and saliva conductivity and hematocrit was negligible (*pC* = −0.00, confidence interval = −0.00 – −0.00, and *pC* = −0.00, confidence interval = −0.00 – 0.00, respectively, Supplementary Figure S1).

### 3.3 The association of disease with serum biochemistry parameters

Thirty calf days were used to generate a balanced data set across the three disease status categories (i.e. 10 healthy days, 10 NCD-H days, and 10 NCD-D days). There were four sets of male beef calves, three sets of female beef calves and three sets of female dairy calves. The descriptive statistics of the continuous calf variables and the serum biochemistry parameters are shown in Supplementary Tables S4, S5.

#### 3.3.1 Serum proteins

There was no difference in STP between the three groups (compared to healthy calves: NCD-H: *p* = 0.87 and NCD-D: *p* = 0.53, Table 3).

**Table 3:** The association of neonatal calf diarrhea and hydration status with serum protein parameters in artificially reared calves.

Variable	Factor	Level	Number of calves^b^	Estimate	Confidence interval	Degrees of freedom	*t-*value	*P-*value
Serum total protein (g/L)	Disease	Healthy	10	Reference	Reference	2	Reference	Reference
		NCD-H^a^	10	−0.54	−7.01 – 5.93		−0.171	0.865
		NCD-D^a^	10	−2.02	−8.49 – 4.45		−0.641	0.527
Albumin (g/L)	Disease	Healthy	10	Reference	Reference	2	Reference	Reference
		NCD-H	10	−0.14	−2.00 – 1.71		−0.160	0.874
		**NCD-D**	**10**	**3.35**	**1.47–5.23**		3.683	**0.001**
	Sex	Female	18	Reference	Reference	1	Reference	Reference
		Male	12	1.48	−0.38 – 3.33		1.645	0.114
	Sire breed-type	Beef	21	Reference	Reference	1	Reference	Reference
		Dairy	9	1.76	−0.24 – 3.75		1.820	0.082
	**Age**		**30**	**0.28**	**0.09–0.47**	**1**	**2.986**	**0.007**
	Age at inclusion into the group pen		30	1.05	−0.34 – 2.45	1	1.559	0.133
Globulin (g/L)	Disease	Healthy	10	Reference	Reference	2	Reference	Reference
		NCD-H	10	0.33	−6.95 – 7.61		0.094	0.926
		NCD-D	10	−4.58	−11.82 – 2.65		−1.302	0.204
	Age		30	−0.45	−1.14 – 0.24	1	−1.340	0.192
IgG (g/L)	Disease	Healthy	10	Reference	Reference	2	Reference	Reference
		NCD-H	10	0.49	−9.69 – 10.67		0.098	0.923
		NCD-D	10	−9.16	−19.28 – 0.96		−1.860	0.074
	Age		30	−0.78	−1.74 – 0.19	1	−1.653	0.110

The results shown are from the final linear models. Variables shown in bold have *p* < 0.05.

a NCD-H: neonatal calf diarrhea-hydration status normal; NCD-D: neonatal calf diarrhea-dehydrated.

b As each calf was only included once in this dataset the number of calves and the number of calf days are equivalent.

**Figure 3:**
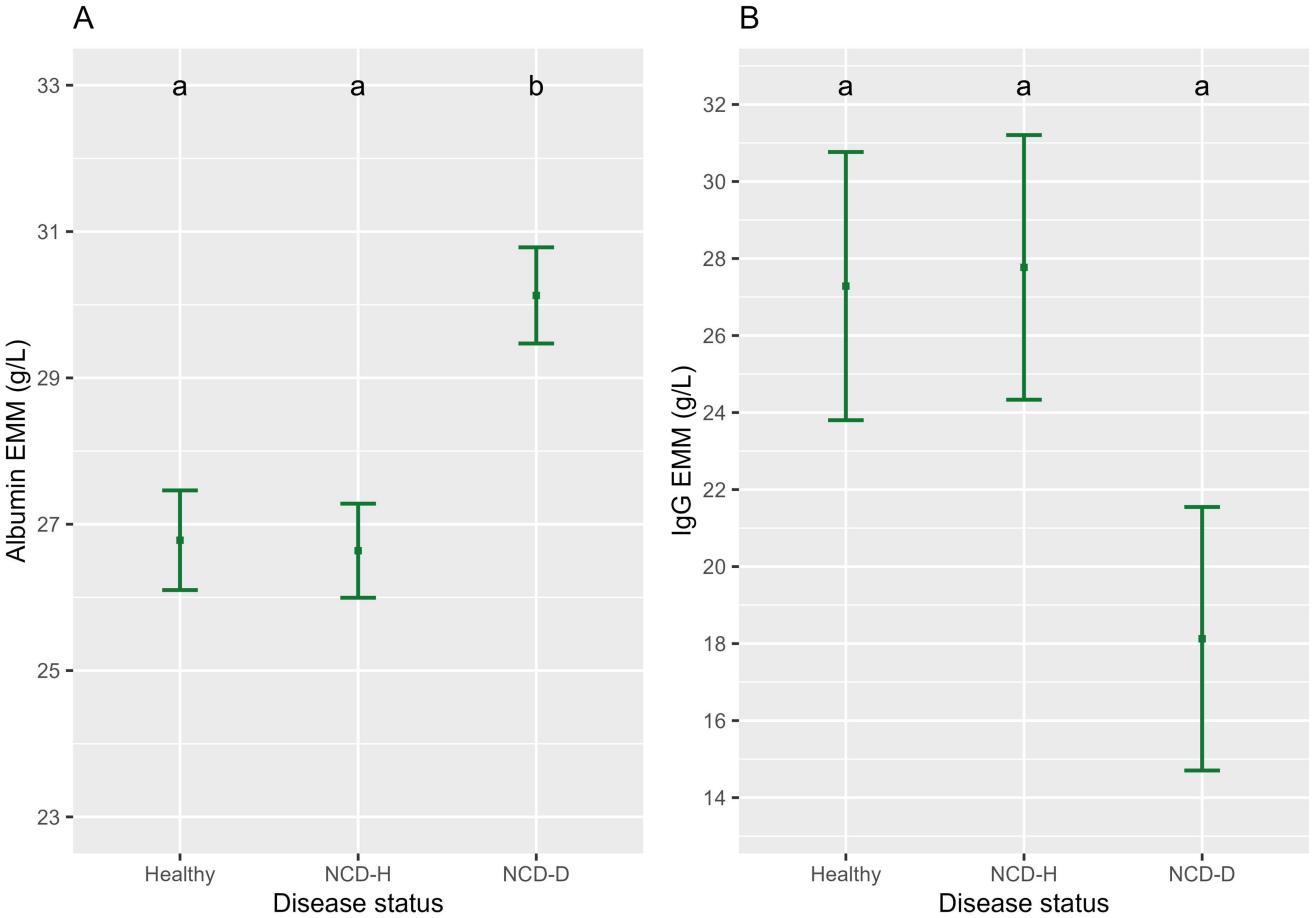
The association of neonatal calf diarrhea and hydration status with **(A)** albumin and **(B)** IgG. Plots and error bars denote the estimated marginal means (EMM) and corresponding standard errors calculated from the final model. Differing letters indicate statistically significant differences. NCD-H: neonatal calf diarrhea-hydration status normal; NCD-D: neonatal calf diarrhea-dehydrated.

Albumin was highest in NCD-D calves compared to their healthy or NCD-H counterparts (*p* < 0.01, and *p* < 0.01, respectively, Figure 3B). There was no difference between NCD-H and healthy calves (*p* = 0.99, Figure 3A).

There was no difference in globulin concentrations between the three groups (compared to healthy calves: NCD-H: *p* = 0.93; NCD-D: *p* = 0.20, respectively, Table 3).

IgG was not different between the three groups (when compared to healthy calves, NCD-H; *p* = 1.00, NCD-D; *p* = 0.17, Figure 3B).

Older calves had a higher level of albumin than their younger counterparts (*p* < 0.01, *t* = 2.986, DF = 2, Table 3). Age was included in the final models for albumin, globulin and IgG as it improved model fit (*p* = 0.08, *p* = 0.19 and *p* = 0.11 respectively, Table 3). Sex and age at inclusion into the group pen were included in the final model for albumin as they improved the model fit (*p* = 0.11 and *p* = 0.13 respectively, Table 3).

#### 3.3.2 Dehydrated calves had a lower strong ion difference

**Figure 4:**
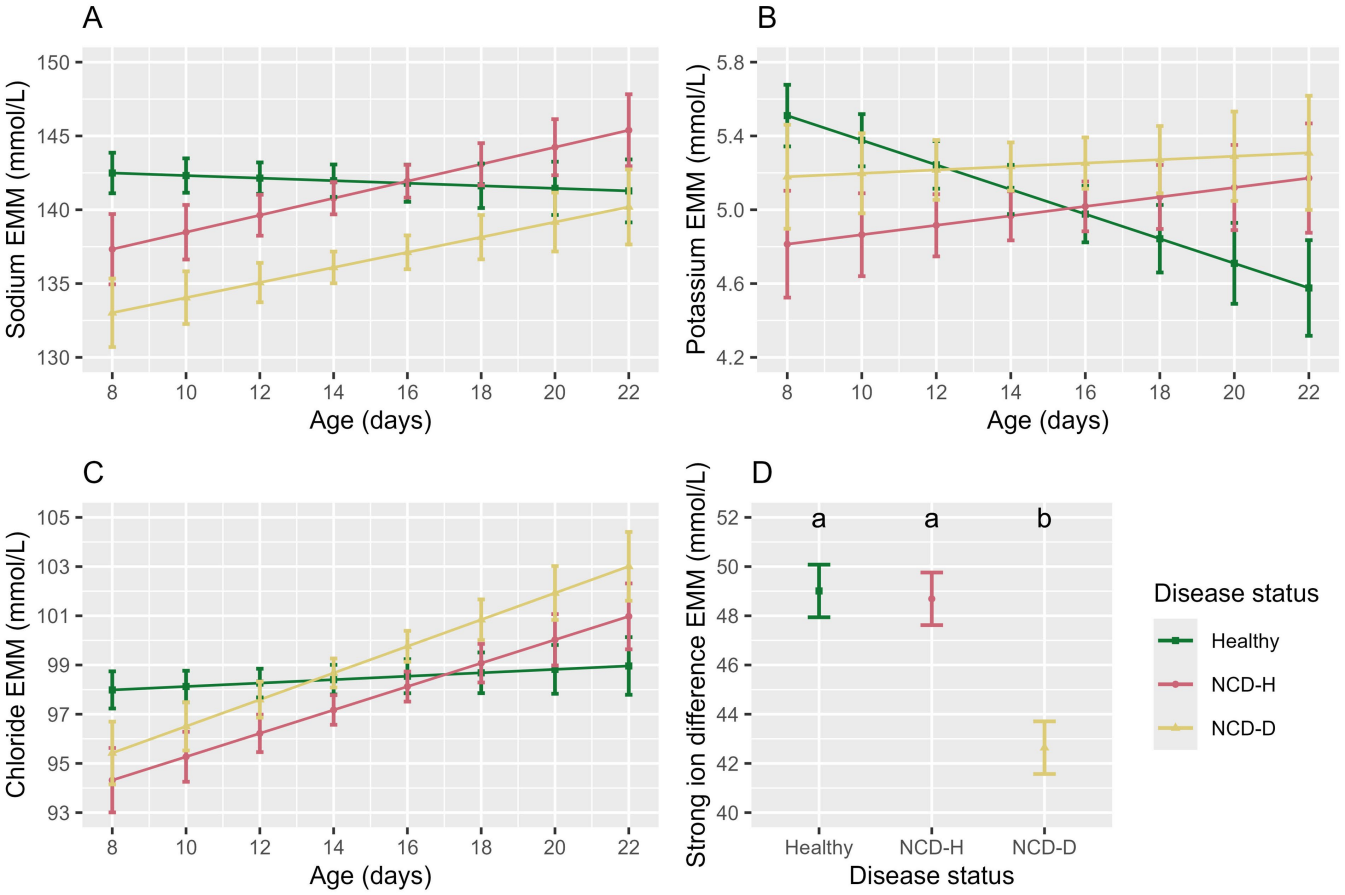
The association of disease status and age with the electrolyte parameters in the balanced data set. Plots and error bars denote estimated marginal means (EMM), and corresponding standard errors calculated from the final models for; **(A)** sodium, **(B)** potassium, **(C)** chloride, **(D)** strong ion difference. Differing letters indicate statistical significance. NCD-H: neonatal calf diarrhea-hydration status normal; NCD-D: neonatal calf diarrhea-dehydrated. Each disease group consists of 10 calves.

Calves with NCD-D had the lowest sodium concentration (compared to healthy calves: *p* < 0.01, compared to NCD-H calves: *p* = 0.01, Figure 4A). Sodium concentrations did not differ between NCD-H and healthy calves (*p* = 0.73, Figure 4A). Age and the interaction between age and disease were included in the final model as they improved model fit (*p* = 0.66 and *p* = 0.12, respectively, Figure 4A).

Potassium was lowest in NCD-H calves (when compared to healthy calves: *p* = 0.04, Table 4), however this was no longer true in the pairwise comparison (*p* = 0.73, Figure 4B). There was no difference between NCD-D calves and healthy or NCD-H calves (*p* = 0.78 and *p* = 0.34 respectively, Figure 4B). Younger calves had the highest serum potassium (*p* = 0.010, Figure 4B). There interaction between age and disease was included as it improved model fit (*p* = 0.08, Figure 4B).

**Table 4:** The association of neonatal calf diarrhea and hydration status with serum electrolytes artificially reared calves.

Variable	Factor	Level	Number of calves^b^	Estimate	Confidence interval	Degrees of freedom	*t*-value	*P*-value
Sodium (mmol/L)	Disease	Healthy	10	Reference	Reference	2	Reference	Reference
		NCD-H^a^	10	−10.46	−21.65 – 0.73		−2.648	0.066
		**NCD-D** ^**a**^	10	**−14.26**	**−25.38 – −3.14**		**−1.929**	**0.014**
	Age		30	−0.09	−0.49 – 0.32	1	−0.444	0.661
	Age*Disease							0.120
Potassium (mmol/L)	Disease	Healthy	10	Reference	Reference	2	Reference	Reference
		**NCD-H**	10	**−1.44**	**−2.80 – −0.07**		**−2.177**	**0.040**
		NCD-D	10	−0.94	−2.29 – 0.41		−1.455	0.164
	**Age**		**30**	**−0.07**	**−0.12 – −0.02**	**1**	**−2.187**	**0.010**
	Age*Disease							0.079
Chloride (mmol/L)	Disease	Healthy	10	Reference	Reference	2	Reference	Reference
		**NCD-H**	10	**−6.92**	**−13.07 – −0.77**		**−2.321**	**0.003**
		**NCD-D**	**10**	**−6.34**	**−12.45 – −0.23**		**−2.143**	**0.042**
	Age		30	0.07	−0.15 – 0.29	1	0.650	0.522
	Age*Disease							**0.039**
Strong ion difference (mmol/L)	Disease	Healthy	10	Reference	Reference	2	Reference	Reference
		NCD-H	10	−0.32	−3.42 – 2.78		−0.212	0.834
		**NCD-D**	10	**−6.30**	**−9.47 – 3.27**		**−4.001**	**<0.001**

The results shown are from the final linear models. Variables shown in bold have *p* < 0.05.

a NCD-H: neonatal calf diarrhea-hydration status normal; NCD-D: neonatal calf diarrhea-dehydrated.

b As each calf was only included once in this dataset the number of calves and the number of calf days are equivalent.

Chloride was lowest in NCD-H calves (when compared to healthy calves, *p* = 0.03, Table 4). Chloride was also lower in NCD-D calves compared to their healthy counterparts (*p* = 0.04, Table 4). However no pairwise comparisons of estimated marginal means were significant (*p* > 0.05). Age was not associated with serum chloride (*p* = 0.52, Figure 4C). However, there was an interaction between disease and age (*p* = 0.04, Figure 4C).

The SID was lowest in NCD-D calves (Healthy: *p* < 0.001, NCD-H: *p* < 0.01, Figure 4D). Healthy calves and NCD-H calves were not different from each other (*p* = 0.98, Figure 4D).

#### 3.3.3 Saliva parameters were not associated with strong ion difference

To explore the association between saliva parameters and metabolic acidosis, the Lin’s concordance correlations between saliva parameters and SID were calculated. Neither saliva conductivity or saliva pH were associated with SID (*pC* = −0.00, confidence interval = −0.00 – −0.01, and *pC* = −0.00, confidence interval = −0.00 – 0.00, respectively, Supplementary Figure S2).

## 4 Discussion

This study aimed to assess the relationship of saliva conductivity and pH with the clinical and biochemical parameters of calves suffering from neonatal calf diarrhea (NCD). Saliva conductivity was not associated with NCD with or without dehydration. Saliva pH was associated with NCD with or without dehydration. The Lin’s concordance correlation coefficient with hematocrit or strong ion difference (SID) was negligible, however. Dehydrated calves had a higher hematocrit than calves with NCD without dehydration or healthy calves. Calves with NCD and no dehydration had a lower PTP than healthy or dehydrated calves. Albumin was higher in dehydrated calves. There was no effect of disease with or without dehydration on STP, globulin or IgG. Calves with NCD and dehydration had the lowest sodium and SID.

The incidence of disease in this study was 77 per 100 calves under 25 days of age, this was higher than previously reported in the UK by Johnson et al. (2) (mean 48 per 100 calves in the first 10 weeks of life, range: 24–74) and Johnson et al. (5) (65 per 100 calves in the first 8 weeks of life), this may be due to the daily monitoring of the calves, whereas the previous studies monitored weekly. Previous studies that have carried out daily monitoring have recorded incidence rates of 85 per 100 calves under 28 days of age (6) and 97 per 100 calves under 49 days of age (7). While NCD is a major cause of mortality in calves (4) there were no cases of mortality due to NCD in this study. This is most likely due to early and aggressive oral rehydration therapy when dehydrated calves were identified. It is worth noting however, that the dehydrated calves were all identified by the research staff prior to them being identified by the farm staff and thus treatment was initiated earlier than it would have been in a normal farm situation.

Saliva conductivity was of interest as it has been previously shown to be correlated with serum osmolality in humans (28). The ionic concentration of the fluid affects the conductivity of the fluid in a non-linear function, the use of conductivity as a proxy for osmolality is incorporated into some urinalysis machines (48). Interestingly, previous work on saliva osmolality in humans showed an association between saliva osmolality and body water in humans who have exercised, but not in humans that had undergone passive dehydration (24). Ely et al. (49) found that saliva osmolality in humans was affected by a brief water rinse of the mouth, although the effect lasted less than 15 min. It was not possible to control whether calves had accessed the milk or water within 15 min prior to sampling and so this cannot be excluded as potentially having affected the results of this study. While this study did not identify an association between diarrhea with or without dehydration and saliva conductivity, the limited number of dehydrated calves (*n* = 10) with each only scored as dehydrated for 1 day, means that we cannot conclusively exclude an association between saliva conductivity and moderate to severe dehydration in neonatal calves. In humans, saliva has a role in buffering the oral environment (50). However, in ruminants, far higher levels of sodium, bicarbonate and phosphate are found than in monogastric animals. Saliva is also continuously produced and is produced at high volumes, this is due to saliva having a key role in the buffering of the rumen (51). These differences in composition may explain why the results seen here were different to those seen in previous human studies.

Saliva pH was lower in calves with diarrhea. This is consistent with the presence of concurrent metabolic acidosis in calves with NCD. Metabolic acidosis is caused by an increase in D-lactate caused by fermentation of mal-absorbed carbohydrates in the colon, an increase in l-lactate due to dehydration and decreased tissue perfusion and the loss of bicarbonate in the feces (52). Sodium may also be lost in feces (32). A previous study found that the pH of whole blood was negatively correlated with disease severity (34). In this study there was no difference between dehydrated and normally hydrated calves with NCD, this may be due to the small number of dehydrated calves and warrants further investigation. However, Lorenz (53) previously found no correlation between d-lactic acidosis and dehydration. Age was not associated with saliva pH, which is consistent with the findings of previous authors who have reported that saliva pH increases between 8 and 50 days of age, but not between 8 and 36 days of age (36). Another factor to consider is that the saliva pH may be altered by the nutritional status of the calf which may become inappetent with NCD, further research could analyze the relationship between milk intake and saliva pH.

Hematocrit is traditionally used to measure dehydration. The hematocrit results seen in this study were as expected, with hemoconcentration evident in the dehydrated calves. Hematocrit is an important prognostic indicator, and has been shown to be higher on admission to hospital in diarrheic calves that died, when compared to those that survived (54). Dehydration in this study was classified using the return of the skin tent as it has been shown to be associated with changes in hydration status in the absence of a change in hematocrit or total protein in young calves (38). Belgian Blue cows have been shown to have a higher hematocrit than Holstein Friesian cows (55), which is consistent with the increased hematocrit in beef-sired calves in this study. However, this difference was not seen in Dillane et al. (56) who compared the hematocrit of dairy and beef calves. The lower hematocrit in males calves in this study is consistent with that of previous studies for both beef (56) and Holstein calves (57).

The PTP can be used to aid in interpretation of hematocrit results. PTP in this study was lower in hydrated calves with diarrhea but not in dehydrated calves with diarrhea. This reduction in the PTP of diarrheic calves is consistent with the results of Kabu et al. (58) and Hildebrandt et al. (59), however neither study differentiated between dehydrated and non-dehydrated calves. The serum protein results however are slightly contradictory with no affect of disease on STP, globulin or IgG seen. Albumin levels were higher in the dehydrated calves. It is not clear why these findings are contradictory and further analysis of serum proteins in a greater number of calves is required to further understand these results.

The reduction in potassium in calves with diarrhea but not dehydration was an interesting finding, although it was not seen in the pairwise comparisons. While hypokalemia has been identified in calves undergoing treatment for dehydration (60), all samples were taken in the morning prior to any treatment taking place. Much of the literature in this area relates to hospitalized calves and thus pre-selects severe cases, e.g., Trefz et al. (61) or experimentally induced metabolic acidosis to test the efficacy of treatments, e.g., Schwedhelm et al. (62). In contrast, this study focused on spontaneous occurring disease of all severities by following calves in their normal environment. Previous research has found total protein to be weakly correlated with potassium (*r_P_* = 0.46) so it may be that this finding is linked to the reduction in protein seen in the calves with diarrhea but not dehydration (61). Further work on calves with mild-to-moderate disease is needed to explore the significance of these potassium results. The association between increasing age and reducing serum potassium concentration is consistent with the findings of Dillane et al. (56).

Strong ion difference (SID) was lower in calves with diarrhea and dehydration. This means that calves have lost a greater number of cations when compared to anions and is indicative of metabolic acidosis. Many studies have used measures such as anion gap and extracellular base excess to examine acid–base disturbances in calves (32). This was not possible in this study due to budgetary constraints. SID was chosen for use in this study, as it is commonly used as a proxy for acid–base balance and has been associated with changes in both the suckle reflex and posture in young calves with NCD (32). SID has also been shown to be correlated with lactate in calves with diarrhea and no other clinical signs (63).

Some studies have not clearly differentiated between diarrheic calves with or without dehydration. Hildebrandt et al. (59) found no changes in sodium, potassium or chloride in diarrheic calves, but found a difference in the anion base excess. Interestingly, Trefz et al. (17) found an association between survival and sodium and chloride concentrations, however clinical signs had a greater association with survival. Another study found that calves with diarrhea that died had a higher hematocrit, higher levels of sodium and chloride but no difference in SID or potassium (54). Sayers et al. (34) used a clinical assessment scoring system designed by the research farm and its veterinary surgeons that included signs of dehydration. In that study, disease severity was significantly correlated with sodium, SID, total hemoglobin, and blood pH, but not with potassium or chloride. This concurred with the results for hematocrit, SID, potassium and chloride of this study, but not those of sodium.

In addition to differences in disease severity between studies, NCD is caused by a range of pathogens often acting in concert, which may impact differently on the anion gap and extracellular base excess of calves between studies. In this study the predominate pathogen was Cryptosporidium (62 cases), with Bovine Rotavirus, Bovine Coronavirus and *E. coli* K99, also being identified. Fourteen cases had multiple pathogens identified. *E. coli* K99 was not thought to be significant as the calf in question was 18 days old and this pathogen usually affects calves less than 4 days of age (64). Cryptosporidium infection causes villous atrophy in the small intestine leading to malabsorption, with increased secretion of chloride and bicarbonate and reduced absorption of sodium chloride (65). Bovine Rotavirus and Bovine Coronavirus also cause damage to the villi with decreased absorption of sodium chloride and water (66). The number of calves in this study does not allow for analysis of the biochemical differences between pathogens, or comparisons of pathogen prevalence between the dehydrated and normally hydrated NCD groups.

The NCD was associated with saliva pH in this study. The Lin’s concordance correlation between either hematocrit or SID and saliva pH was negligible, however. This suggests that the mechanism by which saliva pH changes in NCD requires further research. It is possible that saliva pH can be used “pen side” by veterinary surgeons or farm staff when assessing a calf on farm to allow them to choose an appropriate course of treatment. This would allow prompt and appropriate treatment to be administered and thus reduce the risk of mortality. Historically, assessment of hydration status or acid–base balance has required access to equipment that is not possible to carry on to commercial farms. The pH meter used in this study was designed to be portable. There is a need for further research as to whether saliva pH cut-offs can be ascertained at which, e.g., intravenous fluids are indicated rather than oral rehydration solution. Saliva pH could be added to decision trees such as that developed by Trefz et al. (67).

The methods described in this study require a centrifuge for saliva separation and had a one-minute sampling time. This study was designed as proof of concept, and this method would not be practical for on-farm use. In future, it may be possible to measure saliva pH at the milk feeding teat to allow early detection of disease. A further key area of development will be to explore the within-individual variation of saliva pH as this will have a large effect on its use for daily monitoring. Early detection allows early intervention with oral rehydration solution, this will not only improve calf welfare but will improve outcomes and may reduce the need for antibiotics. The automatic measuring of saliva pH may also allow it to be combined with other measures such as feeding behavior to increase accuracy. Accuracy is essential in these systems to prevent the labor cost of unnecessary interventions and incorrect alerts.

## 5 Conclusion

Reductions in saliva pH are associated with NCD, but further work is required to ascertain the physiological mechanism. Work including evaluating a greater number of dehydrated calves is needed to ascertain whether saliva pH can differentiate between different levels of disease severity. Saliva conductivity was not associated with NCD, regardless of hydration status. Changes in blood parameters of dehydrated calves were consistent with previous studies. Further investigation of changes in potassium and serum proteins in NCD with and without dehydration is warranted. Saliva pH has potential as a novel indicator of diarrhea in calves.

## Data Availability

The raw data supporting the conclusions of this article will be made available by the authors, without undue reservation.
