# Supplementary material for: Salivary pH, but not conductivity, is an indicator of diarrhea in neonatal calves
Source: Front Vet Sci. 2024 Dec 18;11:1483890. doi: 10.3389/fvets.2024.1483890 (PMC11688809; doi:10.3389/fvets.2024.1483890)
Supplement: Supplementary file 1 [file Data_Sheet_1.DOCX]

Supplementary Material

# Supplementary Data

Table 1 Samples partitioned by disease status, sire breed type and sex

|  | Sire breed type | | | |
| --- | --- | --- | --- | --- |
|  | Beef | | Dairy | |
| Sex | Female | Male | Female | Male |
| Healthy | 72 | 66 | 18 | 8 |
| NCD-H | 119 | 129 | 45 | 21 |
| NCD-D | 3 | 4 | 3 |  |

Table 2 Descriptive statistics of calf variables, saliva parameters, hematocrit and plasma total protein partitioned by disease status at the time of sample collection

| Variable | Disease status | Mean | Standard Deviation | Median | Minimum | Maximum | Standard error of the mean |
| --- | --- | --- | --- | --- | --- | --- | --- |
| Birthweight (kg) | Healthy | 44.08 | 5.93 | 43.5 | 29 | 63 | 0.47 |
|  | NCD-H | 42.09 | 5.82 | 42 | 28 | 56.5 | 0.34 |
|  | NCD-D | 41.5 | 5.72 | 40 | 36 | 54 | 1.91 |
| Age at inclusion (days) | Healthy | 7.27 | 0.74 | 7 | 6 | 11 | 0.06 |
|  | NCD-H | 7.11 | 0.44 | 7 | 6 | 9 | 0.02 |
|  | NCD-D | 7 | 0 | 7 | 7 | 7 | 0 |
| Age (days) | Healthy | 12.13 | 5.19 | 9 | 7 | 25 | 0.4 |
|  | NCD-H | 14.67 | 3.55 | 15 | 8 | 23 | 0.2 |
|  | NCD-D | 14.6 | 3.57 | 15 | 8 | 20 | 1.13 |
| Saliva conductivity (mS/cm) | Healthy | 7.06 | 1.67 | 6.8 | 3.55 | 12.99 | 0.13 |
|  | NCD-H | 7.31 | 1.61 | 7.16 | 3.75 | 11.65 | 0.09 |
|  | NCD-D | 6.67 | 0.9 | 6.85 | 4.77 | 7.72 | 0.28 |
| Saliva pH | Healthy | 8.17 | 0.23 | 8.18 | 7.41 | 8.76 | 0.02 |
|  | NCD-H | 8.1 | 0.24 | 8.12 | 7.1 | 8.76 | 0.01 |
|  | NCD-D | 7.89 | 0.31 | 7.94 | 7.34 | 8.22 | 0.1 |
| Hematocrit (%) | Healthy | 40.44 | 5.63 | 40 | 25 | 58 | 0.44 |
|  | NCD-H | 40.07 | 6.35 | 40 | 24 | 59 | 0.36 |
|  | NCD-D | 44.4 | 5.41 | 44.5 | 37 | 52.5 | 1.71 |
| Plasma total protein (g/dL) | Healthy | 6.1 | 0.65 | 6.07 | 4.5 | 8.1 | 0.05 |
|  | NCD-H | 5.76 | 0.55 | 5.75 | 4.25 | 7.35 | 0.03 |
|  | NCD-D | 5.94 | 0.6 | 5.82 | 5.15 | 7.2 | 0.19 |


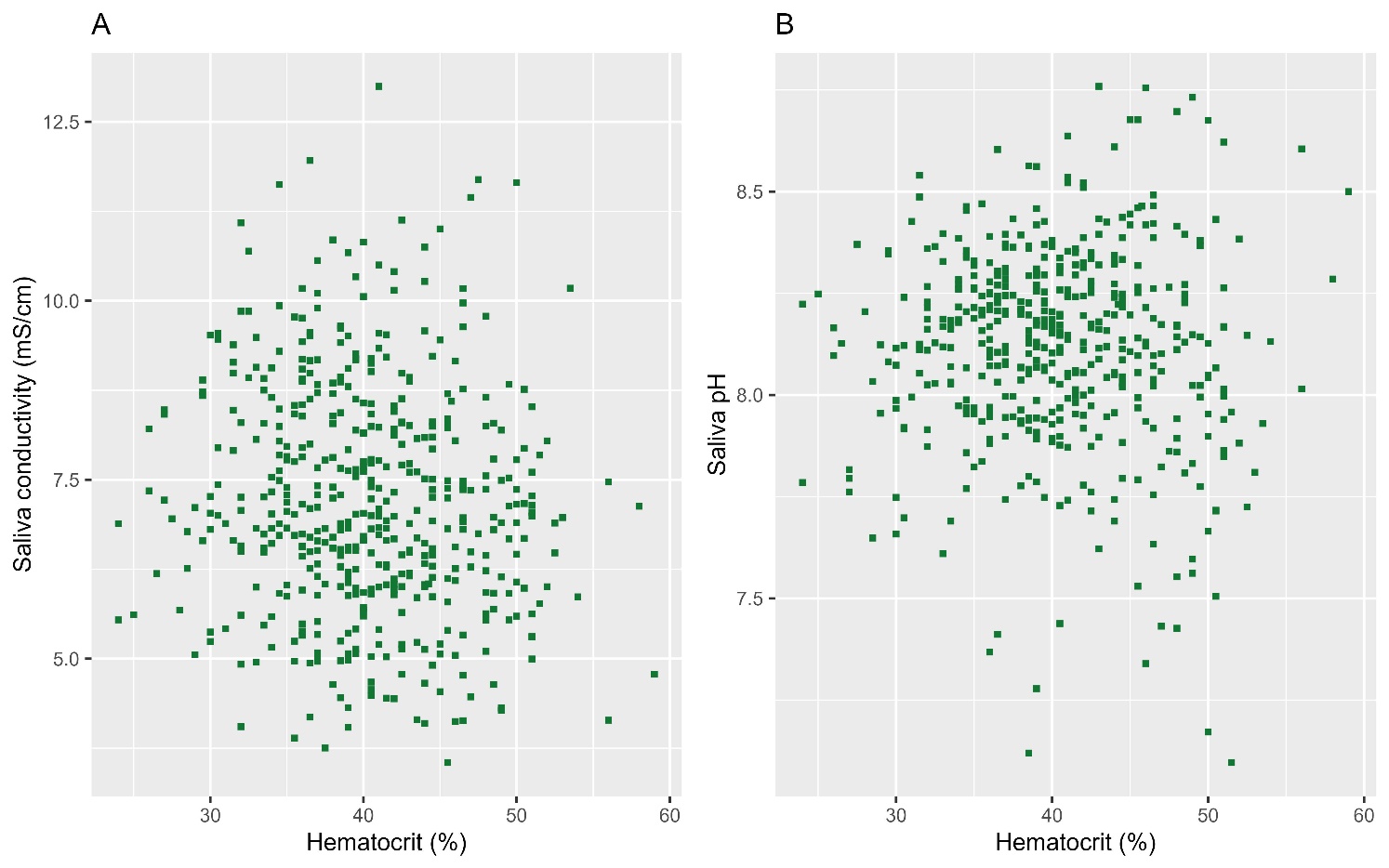


Figure 1 Scatterplots showing the relationships between (A) saliva pH and hematocrit and (B) saliva conductivity and hematocrit .

Table 3 Descriptive statistics of the calf variables partitioned by disease status in a balanced data set of 30 calf days

| Variable | Disease status | Mean | Standard Deviation | Median | Minimum | Maximum | Standard error of the mean |
| --- | --- | --- | --- | --- | --- | --- | --- |
| Birthweight (kg) | Healthy | 42.15 | 5.94 | 42.5 | 33.5 | 51 | 1.88 |
|  | NCD-H | 39.7 | 3.95 | 41 | 32 | 44.5 | 1.25 |
|  | NCD-D | 41.5 | 5.72 | 40 | 36 | 54 | 1.91 |
| Age at inclusion (days) | Healthy | 7.4 | 0.7 | 7 | 7 | 9 | 0.22 |
|  | NCD-H | 7.1 | 0.74 | 7 | 6 | 9 | 0.23 |
|  | NCD-D | 7 | 0 | 7 | 7 | 7 | 0 |
| Age (days) | Healthy | 12.5 | 5.72 | 9 | 8 | 22 | 1.81 |
|  | NCD-H | 14.9 | 3.6 | 15.5 | 10 | 19 | 1.14 |
|  | NCD-D | 14.6 | 3.57 | 15 | 8 | 20 | 1.13 |

**Table 4 Descriptive statistics of the serum biochemistry parameters partitioned by disease status in a balanced data set of 30 calf days**

| Variable | Disease status | Mean | Standard Deviation | Median | Minimum | Maximum | Standard error of the mean |
| --- | --- | --- | --- | --- | --- | --- | --- |
| Serum total protein (g/L) | Healthy | 60.54 | 7.26 | 59.2 | 50.2 | 73.4 | 2.3 |
|  | NCD-H | 60 | 8.79 | 59.3 | 43 | 73.5 | 2.78 |
|  | NCD-D | 58.52 | 4.38 | 57.15 | 53.6 | 66.2 | 1.39 |
| Albumin (g/L) | Healthy | 26.11 | 2.01 | 26.7 | 22.7 | 28.2 | 0.64 |
|  | NCD-H | 26.32 | 1.77 | 26 | 24 | 29.8 | 0.56 |
|  | NCD-D | 29.62 | 2.72 | 28.35 | 27 | 34.4 | 0.86 |
| Globulin (g/L) | Healthy | 58.52 | 4.38 | 57.15 | 53.6 | 66.2 | 1.39 |
|  | NCD-H | 34.43 | 7.91 | 32.95 | 23.8 | 50.4 | 2.5 |
|  | NCD-D | 33.68 | 9.53 | 32.95 | 15.4 | 49 | 3.01 |
| Sodium (mmol/L) | Healthy | 98.3 | 1.42 | 98 | 95 | 100 | 0.45 |
|  | NCD-H | 97.6 | 2.5 | 97 | 94 | 102 | 0.79 |
|  | NCD-D | 99 | 2.75 | 98.5 | 95 | 103 | 0.87 |
| Potassium (mmol/L) | Healthy | 5.21 | 0.52 | 5.15 | 4.4 | 6 | 0.17 |
|  | NCD-H | 4.99 | 0.31 | 5 | 4.5 | 5.4 | 0.1 |
|  | NCD-D | 5.24 | 0.47 | 5.1 | 4.9 | 6.5 | 0.15 |
| Chloride (mmol/L) | Healthy | 98.3 | 1.42 | 98 | 95 | 100 | 0.45 |
|  | NCD-H | 97.6 | 2.5 | 97 | 94 | 102 | 0.79 |
|  | NCD-D | 99 | 2.75 | 98.5 | 95 | 103 | 0.87 |
| Strong ion difference (mmol/L) | Healthy | 49.01 | 3 | 48.25 | 46.2 | 56.8 | 0.95 |
|  | NCD-H | 48.69 | 3.58 | 48.15 | 44.8 | 56.3 | 1.13 |
|  | NCD-D | 42.64 | 3.53 | 42.8 | 37.3 | 46.9 | 1.12 |


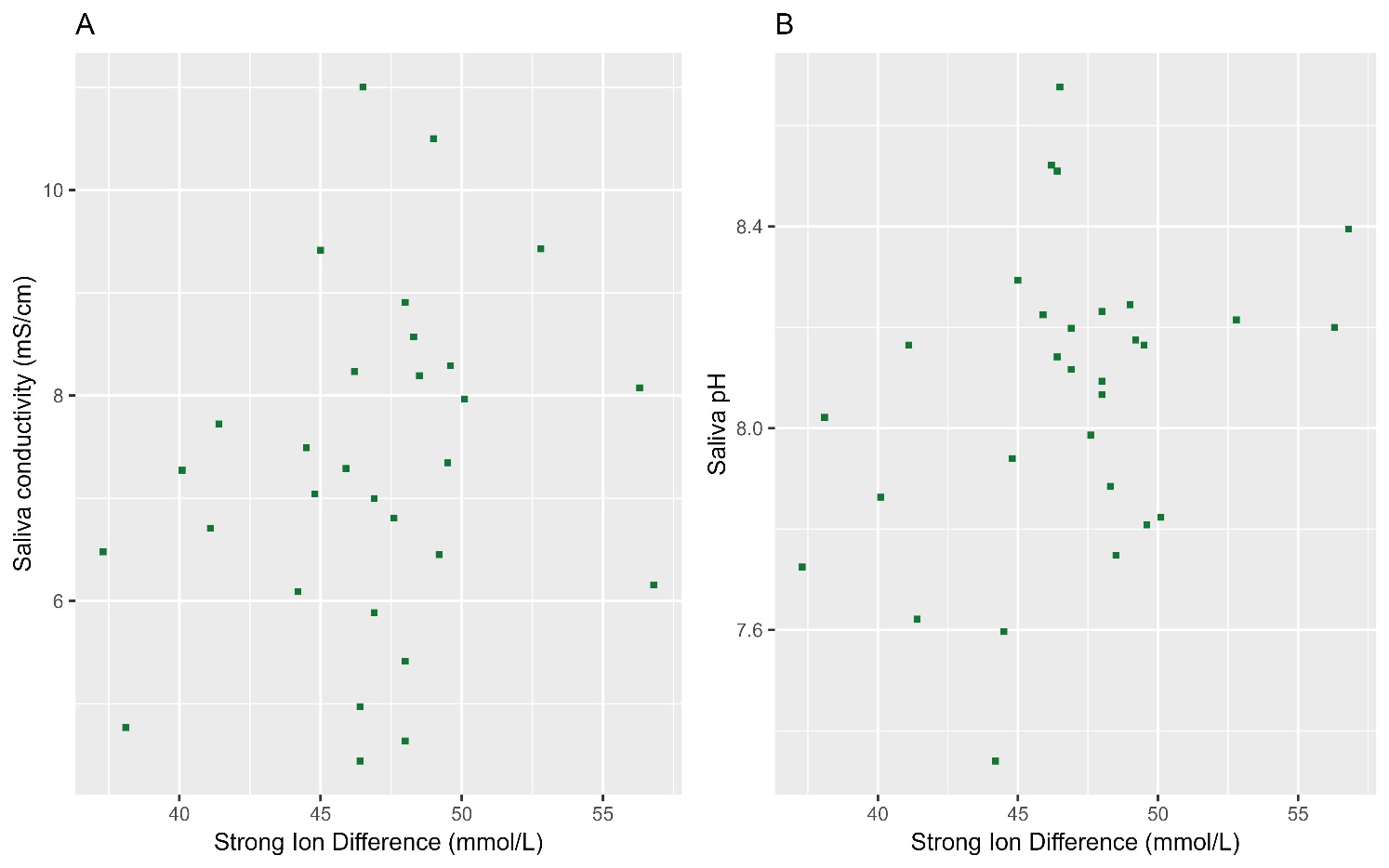


Figure 2 Scatterplots showing the relationships between strong ion difference and (A) saliva conductivity, (B) saliva pH in a balanced dataset of 30 calf days.

# Calf factors associated with differences in saliva and blood parameters in healthy calves

## Methods

The data from the healthy samples was analyzed to check for any known calf factors that may be associated with altered saliva and blood parameters. Univariable general linear mixed models were constructed for hematocrit, PTP, saliva conductivity and saliva pH in turn. The calf identity nested within group was used as a random effect in all models. Age, date of sampling, sire breed-type (beef or dairy), sex, and birthweight were each tested as fixed effects in a univariable model constructed using lmer() in the lme4 (Bates et al., 2015) package of R. The model results were checked using summary() in the lmerTest package (Kuznetsova et al., 2017). Biologically plausible interactions were checked at this stage. Those fixed effects where p<0.2 and interactions where p<0.05 were taken forward for multivariable general linear mixed model building. A forward model building approach was used, adding variables in order of their p values starting with the lowest. The final model was chosen using the corrected Akaike Information Criterion calculated using aictab() (Mazerolle, 2020). The final models were checked using the simulate residuals() and plotQQunif() functions in the DHARMa package (Hartig, 2022). Estimated and p values where calculated using the summary() function in the lmerTest package (Kuznetsova et al., 2017). The confidence intervals were calculated using the confint() function (R Core Team, 2021). The numerator degrees of freedom (NDF) and denominator degrees of freedom (DDF) were calculated using the anova() function (R Core Team, 2021).

## Results

One hundred and sixty-four healthy sampling events from 104 calves were available for analysis. The number of samples within each sex and sire breed-type is summarized in Table 1. Descriptive statistics of the healthy sampling events are summarized in Table 1 of the supplementary materials. The results of the mixed models for saliva conductivity, saliva pH, hematocrit and PTP are shown in Table 6.

Saliva conductivity was higher in male calves when compared to their female counterparts (p = 0.04, Table 5). ~Date was included in the final model as it improved model fit (p = 0.054, Table 5).

Saliva pH had a significant interaction between age and sex (p < 0.05, Table 6, Figure 3). However, neither age or sex were significant individually (p = 0.20, and p = 0.17, respectively, Table 5).

Hematocrit increased over the course of the study period (p = 0.03, t = 2.678, NDF = 1, DDF = 7.56, Table 7). Male calves had a lower hematocrit than their female counterparts (p = 0.0037, t = -2.978, NDF = 1, DDF = 93.624, Table 7). Dairy-sired calves had a lower hematocrit than their beef-sired counterparts (p = 0.0215, t = -2.333, NDF = 1, DDF = 110.358, Table 7).

PTP declined with age (p < 0.0001, t = 5-.234, NDF = 1, DDF = 81.998, Table 7). Calves with a dairy sire had a lower PTP than those with a beef sire (p = 0.0355, t = -2.129, NDF = 1, DDF = 108.499, Table 7).

Table 5 Variables associated with differences in saliva and blood parameters in healthy artificially reared calves. The results shown are outputs from the final linear mixed models. Variables shown in bold have p<0.05.

| **Variable** | **Factor** | **Level** | **Number of calf days** | **Estimate** | **Confidence interval** | **Numerator degrees of freedom** | **Denominator degrees of freedom** | **T value** | **P value** |
| --- | --- | --- | --- | --- | --- | --- | --- | --- | --- |
| Saliva Conductivity  (mS/cm) | Date | | 164^a^ | -0.01 | -0.01 - -0.00 | 1 | 10.644 | -2.171 | 0.054 |
|  | Sex | Female | 90^b^ | Reference | Reference | 1 | 97.731 | Reference | Reference |
|  |  | **Male** | **74^c^** | **0.59** | **0.04 - 1.15** |  |  | **2.100** | **0.038** |
| Saliva pH | Age | | 164 ^a^ | 0.01 | -0.00 - -0.01 | 1 | 140.93 | 1.287 | 0.200 |
|  | Sex | Female | 90 ^b^ | Reference | Reference | 1 | 139.65 | Reference | 0.167 |
|  |  | Male | 74 ^c^ | 0.12 | -0.05 - 0.29 |  |  | 1.388 | 0.742 |
|  | **Age*Sex** | |  |  |  | **1** | **140.32** | **-1.986** | **0.049** |
| Hematocrit  (%) | **Date** | | **164** ^a^ | **0.02** | **0.01 - 0.04** | **1** | **6.5361** | **3.237** | **0.030** |
|  | Sex | Female | 90 ^b^ | Reference | Reference | 1 | **94.253** | Reference | Reference |
|  |  | **Male** | **74** ^c^ | **-2.75** | **-4.50 - -0.82** |  |  | **-2.952** | **0.004** |
|  | Sire breed-type | Beef | 138 ^d^ | Reference | Reference | 1 | 115.270 | Reference | Reference |
|  |  | **Dairy** | **26** ^e^ | **-2.85** | **-5.25 -0.48** |  |  | **-2.406** | **0.022** |
| Plasma Total Protein  (g/dL) | **Age** | | **164** ^a^ | **-0.03** | **-0.05 - -0.02** | **1** | **82.067** | **-5.356** | **<0.001** |
|  | Sire breed-type | Beef | 138 ^d^ | Reference | Reference | 1 | 111.730 | Reference | Reference |
|  |  | **Dairy** | **26** ^e^ | **-0.32** | **-0.62 - -0.03** |  |  | **-2.163** | **0.03** |

^a^ 104 calves, ^b^ 57 calves, ^c^ 48 calves, ^d^ 85 calves, ^e^ 20 calves


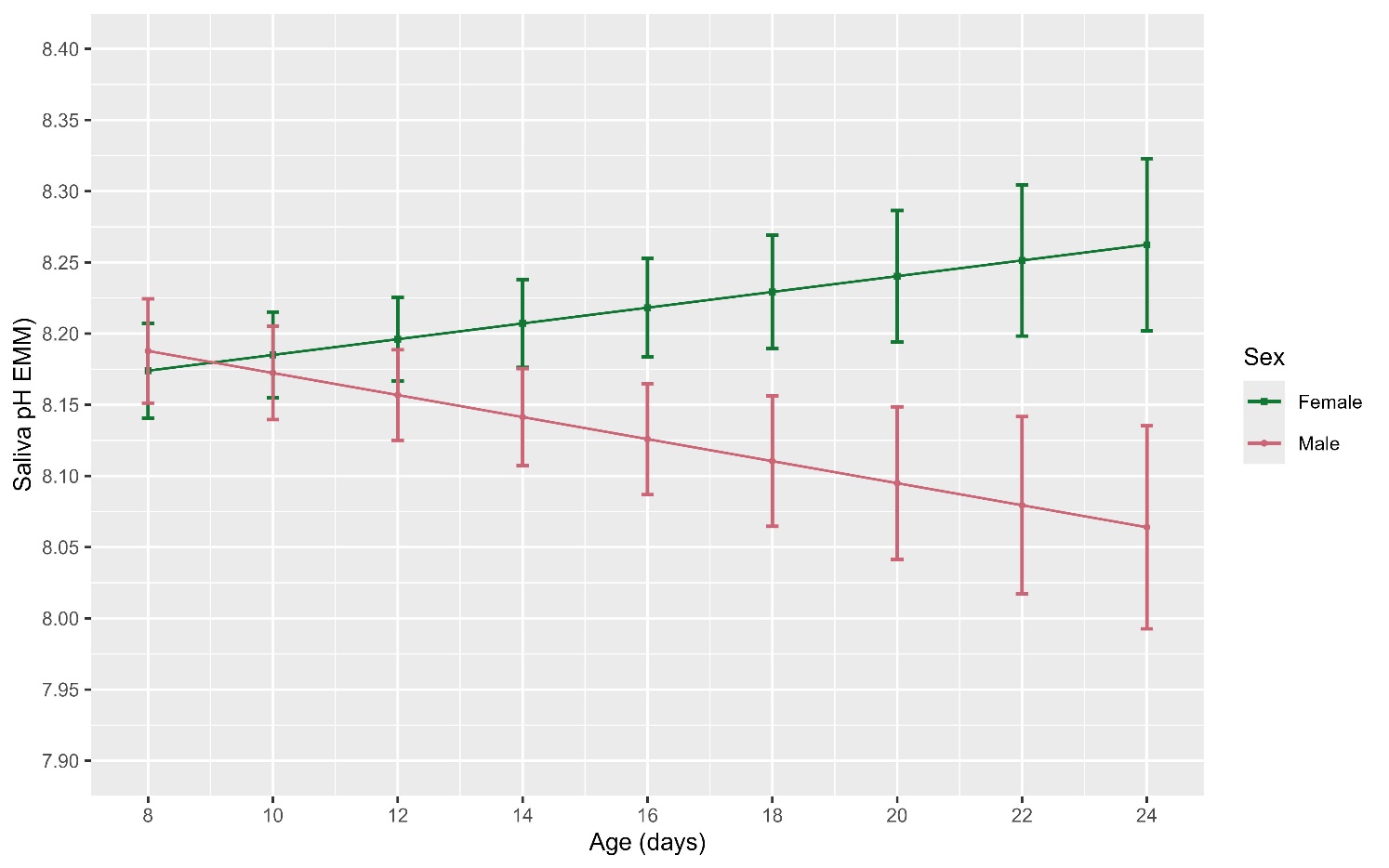


Figure 3 Association of sex and age with saliva pH. Plots and error bars denote the estimated marginal means (EMM) and corresponding standard errors calculated from the final model. Differing letters indicate statistically significant differences.

Bates, D., Mächler, M., Bolker, B., and Walker, S. (2015). Fitting Linear Mixed-Effects Models Usinglme4. *Journal of Statistical Software* 67(1)**,** 1-48. doi: 10.18637/jss.v067.i01.

Hartig, F. (2022). "DHARMa: Residual Diagnostics for Hierarchical (Multi-Level / Mixed) Regression Models".).

Kuznetsova, A., Brockhoff, P.B., and Christensen, R.H.B. (2017). lmerTest Package: Tests in Linear Mixed Effects Models. *Journal of Statistical Software* 82(13)**,** 1-26. doi: 10.18637/jss.v082.i13.

Mazerolle, M. (2020). "AICcmodavg: Model selection and multimodel inference based on (Q)AIC(c)". (<https://cran.r-project.org/package=AICcmodavg>).

R Core Team (2021). "R: A Language and Environment for Statistical Computing". (Vienna, Austria: R Foundation for Statistical Computing).
